# Neuroplasticity and Epilepsy Surgery in Brain Eloquent Areas: Case Report

**DOI:** 10.3389/fneur.2020.00698

**Published:** 2020-07-29

**Authors:** Pedro Jesus Serrano-Castro, Bienvenido Ros-López, Victoria Eugenia Fernández-Sánchez, Natalia García-Casares, Luis Muñoz-Becerra, Pablo Cabezudo-Garcia, Maria José Aguilar-Castillo, Maria Vidal-Denis, Esperanza Cruz-Andreotti, Maria Jose Postigo-Pozo, Guillermo Estivill-Torrús, Guillermo Ibañez-Botella

**Affiliations:** ^1^Neuroscience Unit, Regional University Hospital of Malaga, Málaga, Spain; ^2^Biomedical Research Institute of Malaga, University of Málaga, Málaga, Spain; ^3^Department of Medicine, University of Malaga, Málaga, Spain

**Keywords:** language prehabilitation, epilepsy surgery, eloquent area surgery, neuropshycological measures, Boston test, language functional MRI

## Abstract

**Introduction:** Neuronal plasticity includes changes in any component of the central nervous system in response to intrinsic or extrinsic stimuli. Brain functions that depend on the epileptogenic cortex pose a challenge in epilepsy surgery because many patients are excluded from pre-surgical evaluation for fear of the possible sequelae. Some of these patients may be rescued by enhancing neuronal plasticity with brain neuromodulation techniques.

**Case Report:** We describe a 6-year-old child with refractory focal motor seizures symptomatic to a neuroepithelial dysembryoblastic tumor in the left temporo-parietal region. He underwent limited resection of the lesion in order to avoid sequelae in his language function. A functional study at age of 17 years revealed an overlap of Wernicke's area with the tumor and areas of incipient language reorganization in the contralateral hemisphere. An invasive neuromodulation procedure was designed to enhance neuroplasticity. After craniotomy, he underwent language training and simultaneous electrical inhibition of language using an electrode grid placed over the lesion. The intensity of the language inhibitory stimulus was increased every day to force the use of accessory language areas in the right hemisphere by neuroplasticity.

**Results:** The language of the patient improved for six consecutive days until he was able to speak and understand while undergoing maximum electrical inhibition. The tumor was resected using a cortical mapping guide.

**Discussion:** Application of direct cortical stimulation techniques and language pre-habilitation before epilepsy surgery can be useful to rescue patients excluded from resective surgery, especially young patients with long-term lesions.

## Introduction

In 1894, Santiago Ramón y Cajal was the first to apply the term “plasticity” to the central nervous system at the International Medical Congress held in Rome (1), where he described dynamism or adaptation related to structural neuronal changes in response to external stimuli. Neuronal plasticity is now considered to refer to changes in any component of the central nervous system produced by intrinsic or extrinsic stimuli (2).

Knowledge of neuronal plasticity has expanded over recent decades, through the application of non-invasive electrical or magnetic stimulation procedures to complement conventional cognitive rehabilitation techniques after acquired brain damage (3–6). The main challenges are the evanescence of induced changes due to the distance between application and brain tissue and the interposition of the skull. These limitations may be overcome by using more invasive techniques, such as cortical stimulation mapping (CSM). CSM has long been used to identify eloquent areas in the presurgical study and to demarcate epileptogenic sites. CSM has also confirmed the plastic potential of brains in childhood and adolescence (7, 8). There has been abundant research on the application of CSM in animal models of neuronal plasticity modification (9–11). Functional magnetic resonance imaging (fMRI) studies of humans have also shown that long-term lesions in eloquent areas can permanently modify functional circuits by innate plasticity processes (6, 12, 13).

The prognosis of patients undergoing brain neurosurgery is influenced by the extent of resection, which is limited by the presence of brain functions dependent on the cerebral cortex. This causes many patients to be excluded from functional epilepsy surgery. Some of these patients might be rescued for the only curative treatment currently available if brain neuromodulation techniques could develop their neuronal plasticity. The number of patients who could benefit from such techniques is probably high given that the prevalence of active epilepsy in the world is 6.38/1,000 people (95% CI 5.57–7.30) (14), and of these, ~20–40% behave as refractory to medical treatments (15). Although there are no reliable data in the literature on the percentage of these patients with lesions in eloquent areas, it is known that this is a major clinical problem that has forced the development of various therapeutic strategies in these patients (16).

We report a case in which the neuronal plasticity of language was induced before epilepsy surgery.

## Case Report

We describe the case of a right-handed 6-years-old child with focal motor seizures of the right lower limb and sudden aphasia, without awareness impairment secondary to a space-occupying lesion in the left temporoparietal region. He underwent partial resection of the lesion, which was limited by the need to avoid sequelae in his language function. The pathological study reported WHO grade I neuroepithelial dysembryoplastic tumor (Ki-67 cell proliferation index < 1%). After the surgery, the patient continued with daily epileptic seizures refractory to medical treatment.

At the age of 17 years, a follow-up neuroimaging study showed an increase in the volume of the lesion, and an fMRI scan revealed an overlap of the area of Wernicke with the tumor and areas of incipient functional language reorganization in the homologous contralateral hemisphere.

Since the beginning of his illness, the patient has undergone multiple drug regimens, including oxcarbazepine, valproic acid, lamotrigine, eslicarbazepine acetate, lacosamide clobazam, and brivaracetam, in different rational combinations, without achieving the goal of freedom from seizures.

### Methods

An invasive neuromodulation procedure was designed to enhance neuroplasticity.

Step 1: First awake intraoperative CSM: As the preoperative fMRI showed some transferred language areas to the right hemisphere, a first CSM was performed intraoperatively to confidently assess whether there was or not residual and functional language located over or nearby the tumor. Awake CSM followed left parietotemporal-wide craniectomy over the lesion. Phase-reversal of N20 was first tested. Once Rolandic sulcus was accurately showed up, the motor strip was stimulated while performing electrocorticography, with a monopolar handheld stimulating probe rectangular, monophasic, anodal multipulse (*N* = 7 ISI = 4 [250 Hz]) stimulus, with a duration of 0.2–0.5 ms and up to 25 mA of intensity using a 16-channel neurophysiological intraoperative monitoring device (Protektor by Xtelk®). With the motor threshold, we started the language direct cortical stimulation mapping using the Penfield technique with a handheld bipolar probe with 5 mm between the tips of the probe (biphasic starting positive, at 60 Hz, duration of 1 ms) with an intensity between 2 and 20 mA during 4 sg (*N* = 240 stimuli) using a cortical stimulator (Nimbus®), while the patient was performing motor language tasks: counting numbers days of the week; comprehension tasks: pictures descriptions, and repetition, reading, and witting tasks. As we found, there was some residual language just over the tumor that should be resected. To minimize language deficits, we decided to continue the procedure of prehabilitation and proceed with the placement of 20 subdural grid electrodes.

**Figure 1:**
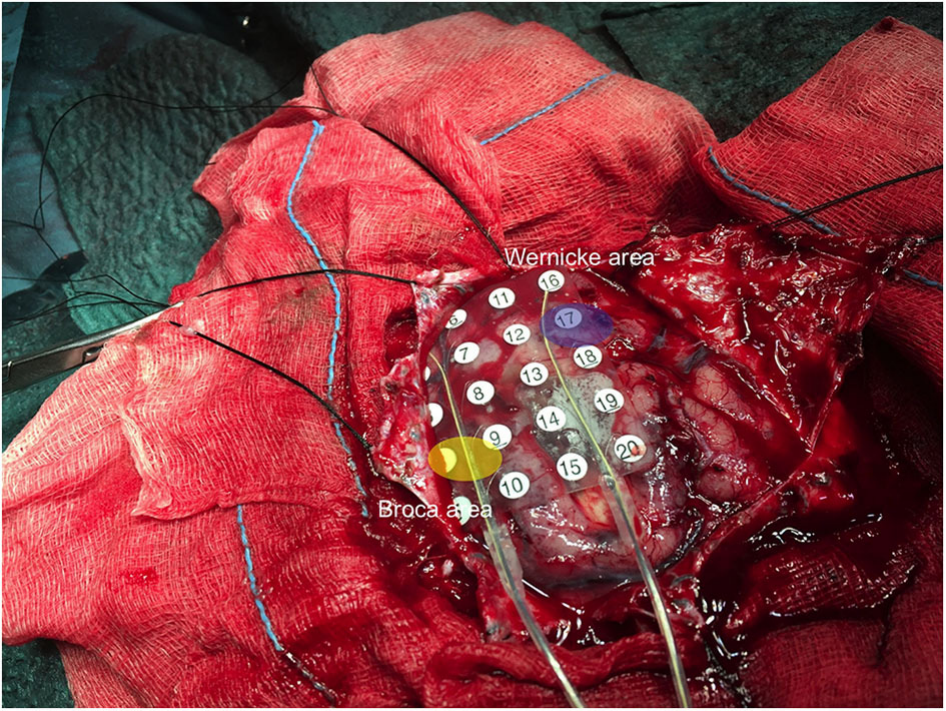
Surgical image during CSM, indicating the sensitive and motor language eloquent areas identified.

Figure 1 depicts the location of functional areas of language; electrode 17 is located on the sensitive area (Wernicke's) and electrode 4 on the motor area (Broca).

Step 2: Language Prehabilitation: 1 day after this surgery, in the patient's room, we performed a cortical stimulation through the 20 subdural grid electrodes, detecting the electrodes that were over Broca and Wernicke areas. Once the target electrodes were identified, we connected them (electrodes 4 and 17) to an external stimulator (Medtronic 3625, Medtronic Ibérica SA) to perform continuous electrical stimulation. The stimulation of these electrodes generated language dysfunction. The parameters used were 130 Hz, 1 ms, and intensity up to 10 V, which was increased daily in steps of 2 V to reach the limit of language function inhibition.

**Figure 2:**
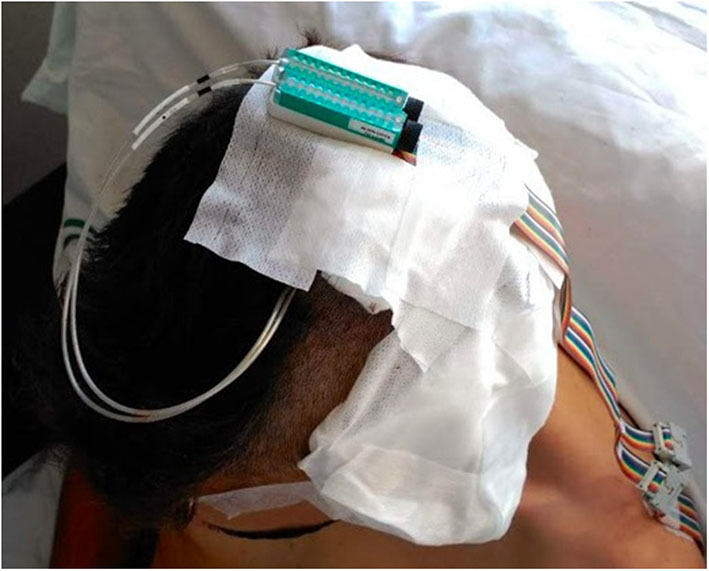
Detail of the position of the external stimulator during step 2.

This stimulation was continuously active for 6 days, with increases or changes between the stimulating electrodes when necessary to reach the inhibition of the language again, as a habituation phenomenon was present (Figure 2).

During this procedure, every day, after adjustment of the intensity of the continuous electrical stimulation, during at least 6 h a day, the neuropsychologist performed an intensive work on the specific deficits of the patient using material from the Spanish version of Psycholinguistic Assessments of Language Processing in Aphasia (PALPA) for the training of spontaneous language and denomination (17) and material from the Barcelona test for the training of understanding, denomination, repetition, reading, and writing (18).

Step 3. Second Awake Cortical Mapping and Definitive Surgery: On the 7th day after the first awake craniotomy, the definitive surgery was done. The second CSM revealed that there was no residual language over the tumor that was completely resected with no further functional deficits.

At 1 month before surgery and again at 3 months post-surgery, the patient underwent a neuropsychological evaluation of language and an fMRI with language paradigms.

The Boston Naming Test is one of the most widely used visual confrontation naming tests to evaluate the lexical and semantic system in aphasic patients (19). We used this test for language evaluation, as it is a widely used test in the preoperative evaluation of epileptic patients and with which our group has extensive experience.

A 3.0 Teslas Philips Intera® MRI (release 2.6) system was used for blood-oxygen-level-dependent (BOLD) signal fMRI acquisition. The scanning session included one T1 structural image for precise anatomical localization of language areas and T2-weighted fast field echo, echo planar imaging (repetition time 3,000 ms, echo time 35 ms, field-of-view 230 mm, and matrix size 80/128 r). Auditory and block design fMRI paradigms (verbal fluency, semantic decision, verb generation, and passive story listening) were performed to determine the eloquent areas of language Broca and Wernicke.

### Ethics

The patient and his parent signed informed consent in the hospital. The study was conducted following the principles of the Declaration of Helsinki (20), with Spanish regulations on biomedical research and with European personal data protection regulations. It was approved (code 0698-N-20) by the institutional ethical committee of our hospital (Comité de Ética de la Investigación provincial de Málaga).

## Results

### Prehabilitation Procedure

The patient improved his linguistic ability for 6 consecutive days after the start of language prehabilitation. On the day before the second surgery, he was able to speak and understand without major deficits despite the application of maximum electrical inhibition to the Wernicke area of the left hemisphere. The tumor was then completely resected with cortical mapping in the awake patient.

### Outcome

The patient has been seizure-free for more than 1 year after the surgery and has returned to his usual academic and social activities. He is currently receiving brivaracetam and eslicarbazepine acetate in descending doses.

### Neuropsychological Evaluations

Neuropsychological language evaluations in our patient showed a progressive deterioration over the 2 years preceding surgical intervention in listening, fluency, denomination, and writing. More severe impairment was observed in some categories explored by the Boston test, including those related to category denomination and especially, written vocabulary and narrative writing, which deteriorated from normal results for his age at 2 years presurgery to very low scores at 1 month presurgery (Table 1).

**Table 1:** Results of the regulated neuropsychological evaluation at 2 years before surgery, 1 month before surgery, and 3 months after surgery.

		**Boston test**				
		**2 years before surgery (Percentile)**	**1 month before surgery (Percentile)**	**Language evolution previous to surgery**	**3 months after surgery (Percentile)**	**Language Evolution after surgery**
Auditive understanding	Word discrimination	60	40	↓	50	↑
	Orders	100	70	↓	100	↑↑
	Complex Ideation material	60	40	↓	70	↑↑
Fluency	Phrase length	100	70	↓	100	↓
	Melodic line	100	70	↓	60	↓
	Grammatical form	100	70	↓	70	=
Recitation		100	100	=	100	=
Repetition	Words	100	100	=	100	=
	Sentences	100	100	=	100	=
Denomination	Naming response	100	70	↓	100	↑↑
	Boston vocabulary test	40	60		70	↑
	Category Denomination	100	50	↓↓	100	↑↑
Reading	Match writing types	100	100	=	100	=
	Match numbers	100	100	=	100	=
	Match drawing-word	40	30	↓	40	↑
	Reading words aloud	100	100	=	100	=
	Reading sentences aloud	100	100	=	100	=
	Understanding sentences spoken aloud	100	100	=	100	=
	Understanding sentences and paragraphs spoken aloud	100	60	↓	100	↑↑
Writing	Shape	100	100	=	100	=
	Choice of letters	100	100	=	100	=
	Motor facility	100	40	↓↓	50	↑
	Basic vocabulary	100	30	↓↓↓	100	↓↓↓
	Regular phonetics	100	100	=	100	=
	Common irregular words	100	100	=	100	=
	Written designation of drawings	100	100	=	100	=
	Narrative writing	100	40	↓↓	80	↑↑

*Percentile values. Red shading: <50 percentile. Orange shading: 50–90 percentile range. Green shading: >90 percentile. ↓↓↓, very important worsening; ↓↓, important worsening; ↓, worsening; =, no changes; ↑, moderate improvement; ↑↑, significant improvement; ↑↑↑, very important improvement*.

These deficits were recovered after the language prehabilitation process and tumor resection. The most important recoveries were observed in areas of auditory understanding, denomination, basic vocabulary, and narrative writing.

From a practical viewpoint, this improvement opened up the possibility of the patient returning to his normal academic life after surgery.

### fRMI

**Figure 3:**
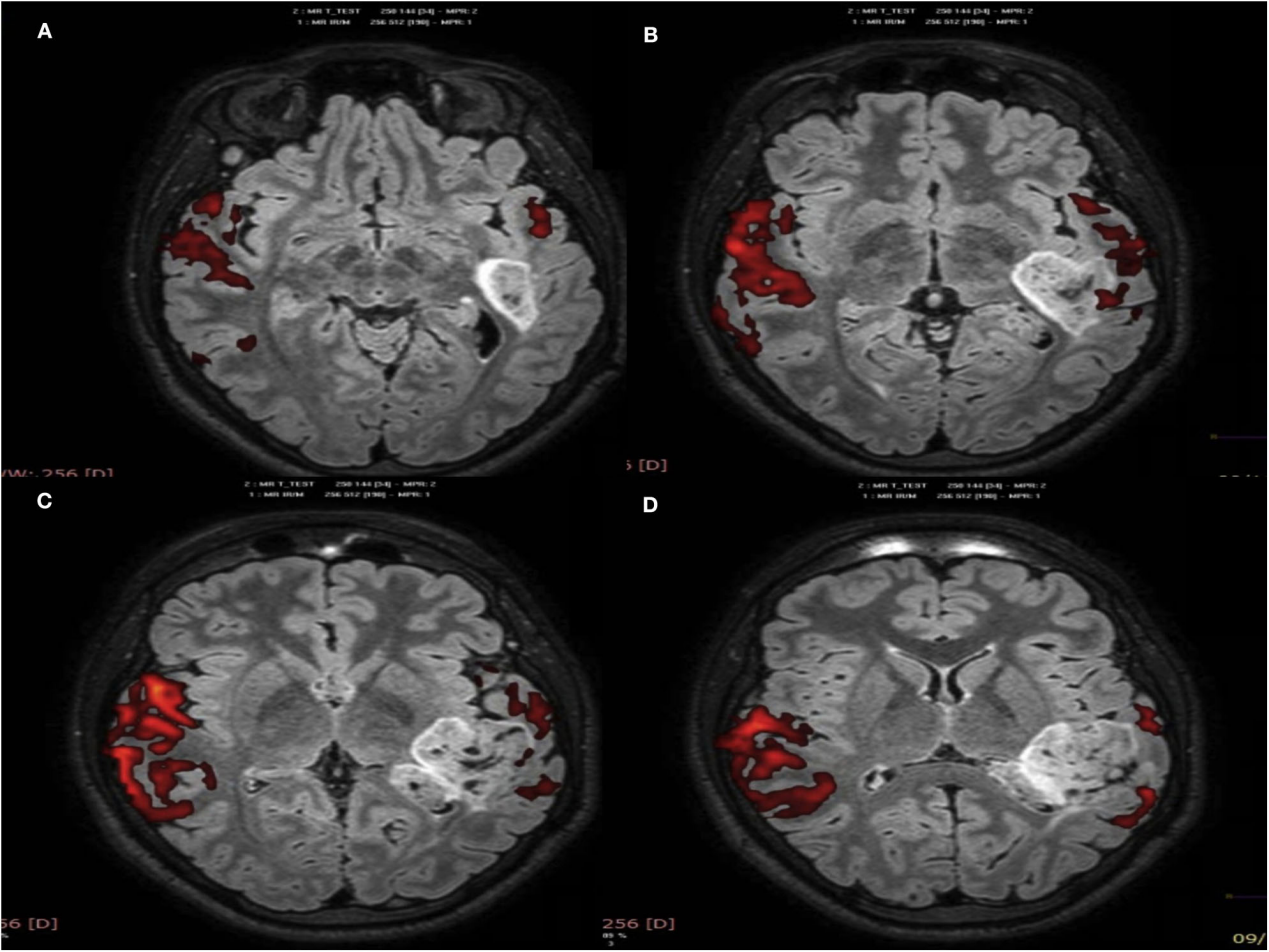
Presurgical fMRI, story passive listening paradigm **(A–D)**: axial brain planes show, in red color, the activation of left temporoparietal areas corresponding to Wernicke's, associative language, and the auditory areas within and surrounding the lesion. Greater activation is shown in the right temporoparietal hemisphere (homologous areas), probably due to neuroplasticity.

**Figure 4:**
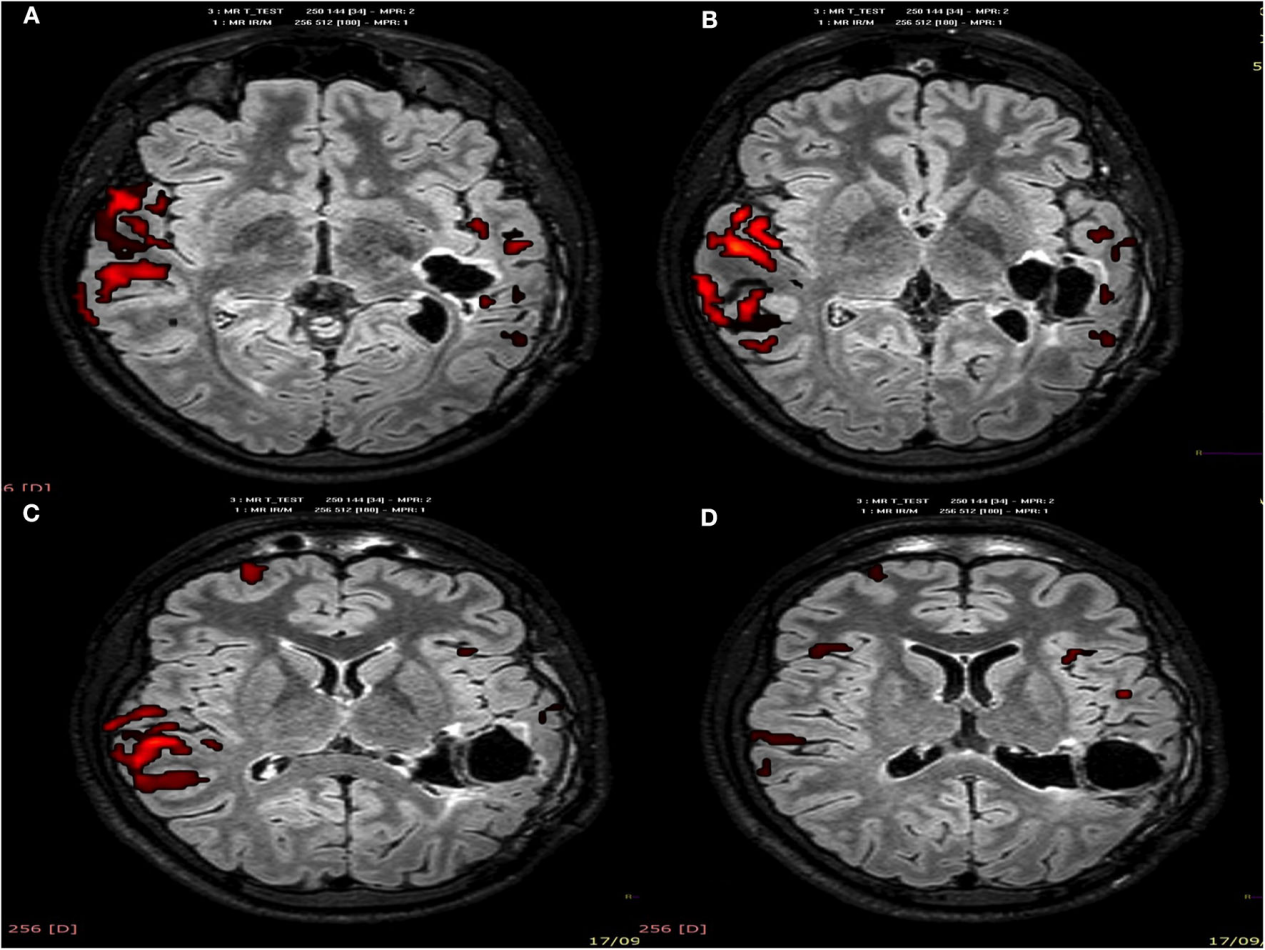
Postsurgical fMRI, story passive listening paradigm **(A–D)**: axial brain planes show, in red color, a decrease activation after surgery of the left temporoparietal areas corresponding to Wernicke's, associative language, and the auditory areas. Greater activation is shown in the right temporoparietal hemisphere (homologous areas), probably due to neuroplasticity.

When comparing fMRI before and after the tumor resection and the stimulation protocol, postsurgical images show decreased activity in the left hemisphere areas and greater activation in the right temporal areas, including the right homologous area of the left Wernicke's. This suggests neuroplasticity in these right eloquent auditory and language areas and could explain the improvements in language comprehension in this patient (see Figures 3, 4).

## Discussion

To our best knowledge, we present the first report of modulation of cerebral plasticity in a patient undergoing epilepsy surgery in language-eloquent areas. In 2016, Rivera et al. (21) described a series of five patients with WHO grade II or III glial lesions in language-eloquent areas who underwent a similar procedure, reporting that it induced an acceleration of neuroplasticity processes. They were older than the present patient, and their lesions were more recent, circumstances that do not favor neuroplasticity. Besides his younger age, our patient had a very long-term lesion, and an intrinsic neuroplasticity process was already underway (see Figure 3). Chronic lesions in the eloquent cortex are known to cause neuroplasticity that results in the cortical reorganization of functional areas (22–24). In this way, patients can develop language-eloquent areas in other parts of the brain, usually in contralateral homologous areas.

We consider that the improvement found between 1 month presurgery and 2 months post-surgery in our patient indicates the implementation of the incipient functional areas of language developed by neuroplasticity in the right hemisphere over the years, similar to previous observations in patients with long-standing lesions in eloquent areas (23, 24).

We propose that functional inhibition of the Wernicke area of the left hemisphere, together with the simultaneous intensive language training, enabled this process. In this line, good outcomes have previously been described for the rehabilitation of stroke sequelae through the inhibition of functional areas and the simultaneous rehabilitation of damaged areas (25, 26).

These results suggest that the prehabilitation of language with this type of procedure can help in the implementation of areas developed by intrinsic neuroplasticity in patients with long-term lesions in language-eloquent areas. The prehabilitation process is probably not capable of transferring functions, as claimed by Rivera et al., but it can implement an area previously developed by intrinsic neuroplasticity.

Many molecular adjustments have been found and may constitute the substrate of neuroplasticity changes induced by electrical neuromodulation. According to recent molecular studies, direct current stimulation produces significant changes in neurotrophic factors, especially on brain-derived neurotrophic factor (BDNF). Thus, variations in BDNF secretion correlated to recovery after direct stimulation in preoperative treatment of pain control and Parkinson's disease (27, 28). Similarly, elevated nerve growth factor (NGF) serum levels in patients with depression have been suggested as adaptive neuroplasticity and associated with cognitive improvement after direct current stimulation (29). Recent works in experimental models have demonstrated that direct current stimulation in the CA1 region of rat hippocampus mediates elevated levels of BDNF in the hippocampus and priming of N-methyl-D-aspartate receptor-dependent long-term potentiation, eliciting metaplastic aftereffects on hippocampal synaptic plasticity. Induced enhancement of long-term potentiation was completely blocked with an antagonist of TrkB, demonstrating the role of BDNF/TrkB signaling in these effects (30). More recent, circulatory microRNAs (miRNAs) have also been involved in neuronal plasticity response in neuropathological conditions, and they may represent a fine-tuning mechanism able to integrate multiple inputs and outputs. In this sense, a very recent analysis from serum profiles and exosomal miRNAs showed genetic pathways involved in neuronal cell proliferation and differentiation significantly enriched with miRNA targets and identified epilepsy-induced peripheral downregulation of miR-15a-5p, miR-34a, miR-106b-5p, and miR-146 (31). Furthermore, electric stimulation of the ventral hippocampal commissure delays the development of epilepsy in a rat model and produces a highly specific regulation of a set of miRNAs implicated in the shape of dendritic spines (32).

The most important study weakness is that it addresses an isolated case, limiting the conclusions that can be drawn. Nevertheless, it opens the way for investigation of an application that could have a major impact on patients with refractory epilepsy who experience a progressive deterioration but cannot currently access epilepsy surgery. On the other hand, there is no established protocol for prehabilitation, and it is possible that outcomes could be improved by applying different parameters.

In conclusion, direct cortical stimulation techniques and simultaneous language prehabilitation may be a useful approach in epilepsy surgery, especially in young patients with long-term lesions who have demonstrated the beginning of function remodeling through intrinsic neuroplasticity.

## Ethics Statement

The studies involving human participants were reviewed and approved by the Institutional Ethics Committees of our hospital (Comité de Ética de la Investigación provincial de Málaga, code 0698-N-20). Written informed consent was obtained from the minor(s)' legal guardian/next of kin for the publication of this case report, including any potentially identifiable images or data included in this article.

## Author Contributions

PS-C, BR-L, VF-S, LM-B, GE-T, and GI-B contributed in the conception and design of the procedure. PS-C, BR-L, VF-S, LM-B, GI-B, PC-G, MA-C, MV-D, EC-A, and MP-P participated in the implementation of the procedure. PS-C wrote the first draft of the manuscript. All authors contributed to manuscript revision, read, and approved the submitted version.

## Conflict of Interest

The authors declare that the research was conducted in the absence of any commercial or financial relationships that could be construed as a potential conflict of interest.

## References

[1] Serrano-Castro PJ Garcia-Torrecillas JM. Cajal's first steps in scientific research. Neuroscience. (2012) 217:1–5. 10.1016/j.neuroscience.2012.05.008 22588002

[2] Cramer SC Sur M Dobkin BH O'Brien C Sanger TD Trojanowski JQ. Harnessing neuroplasticity for clinical applications. Brain. (2011) 134:1591–609. 10.1093/brain/aws017 21482550 PMC3102236

[3] He W Fong PY Leung TWH Huang YZ. Protocols of non-invasive brain stimulation for neuroplasticity induction. Neurosci Lett. (2020) 719:133437. 10.1016/j.neulet.2018.02.045 29476796

[4] D'Agata F Peila E Cicerale A Caglio MM Caroppo P Vighetti S. Cognitive and neurophysiological effects of non-invasive brain stimulation in stroke patients after motor rehabilitation. Front Behav Neurosci. (2016) 10:135. 10.3389/fnbeh.2016.00135 27445730 PMC4919333

[5] Crinion J Price CJ. Right anterior superior temporal activation predicts auditory sentence comprehension following aphasic stroke. Brain. (2005) 128:2858–71. 10.1093/brain/awh659 16234297

[6] Sampaio-Baptista C Sanders ZB Johansen-Berg H. Structural plasticity in adulthood with motor learning and stroke rehabilitation. Annu Rev Neurosci. (2018) 41:25–40. 10.1146/annurev-neuro-080317-062015 29490196

[7] Chou N Serafini S Muh CR. Cortical language areas and plasticity in pediatric patients with epilepsy: a review. Pediatr Neurol. (2018) 78:3–12. 10.1016/j.pediatrneurol.2017.10.001 29191650

[8] Serafini S Komisarow JM Gallentine W Mikati MA Bonner MJ Kranz PG. Reorganization and stability for motor and language areas using cortical stimulation: case example and review of the literature. Brain Sci. (2013) 3:15971614. 10.3390/brainsci3041597 24961623 PMC4061887

[9] Zheng J Liu L Xue X Li H Wang S Cao Y. Cortical electrical stimulation promotes neuronal plasticity in the peri-ischemic cortex and contralesional anterior horn of cervical spinal cord in a rat model of focal cerebral ischemia. Brain Res. (2013) 1504:25–34. 10.1016/j.brainres.2013.01.015 23370004

[10] Cecatto RB Maximino JR Chadi G. Motor recovery and cortical plasticity after functional electrical stimulation in a rat model of focal stroke. Am J Phys Med Rehabil. (2014) 93:791–800. 10.1097/PHM.0000000000000104 24800715

[11] Adkins DL. Cortical stimulation-induced structural plasticity and functional recovery after brain damage. In: Kobeissy FH editor. Brain Neurotrauma: Molecular, Neuropsychological, and Rehabilitation Aspects. Chapter 43. Frontiers in Neuroengineering. Boca Raton, FL: CRC Press/Taylor & Francis (2015). p. 1–38. 26269915

[12] Rutten GJ Ramsey NF van Rijen PC Alpherts WC van Veelen CW. FMRI-determined language lateralization in patients with unilateral or mixed language dominance according to the Wada test. Neuroimage. (2002) 17:447–60. 10.1006/nimg.2002.1196 12482097

[13] Deng X Xu L Zhang Y Wang B Wang S Zhao Y. Difference of language cortex reorganization between cerebral arteriovenous malformations, cavernous malformations, and gliomas: a functional MRI study. Neurosurg Rev. (2016) 39:241–9. 10.1007/s10143-015-0682-7 26564149

[14] Fiest KM Sauro KM Wiebe S Patten SB Kwon C-S Dykeman J. Prevalence and incidence of epilepsy. A systematic review and metaanalysis of international studies. Neurology. (2017) 88:296–303. 10.1212/WNL.0000000000003509 27986877 PMC5272794

[15] Brodie MJ. Diagnosing and predicting refractory epilepsy. Acta Neurol Scand Suppl. (2005) 181:36–9. 10.1111/j.1600-0404.2005.00507.x 16238707

[16] Maesawa S Nakatsubo D Fujii M Iijima K Kato S Ishizaki T. Application of awake surgery for epilepsy in clinical practice. Neurol Med Chir. (2018) 58:442–52. 10.2176/nmc.oa.2018-0122 30249918 PMC6186762

[17] Kay J Lesser R Coltheart M. PALPA: Psycholinguistic Assessments of Language Processing in Aphasia. Hove: Lawrence Erlbaum Associates (1992).

[18] Peña-Casanova J Normalidad, semiología y patología neuropsicológicas. Programa integrado de exploración neuropsicológica. “Test Barcelona.” Barcelona: Masson (1991).

[19] Kaplan E Goodglass H Weintrab S The Boston naming test: Experimental edition (1978). 2nd ed Boston: Kapan & Goodglass; Philadelphia: Lea & Febiger (1978).

[20] World Medical Association World Medical Association Declaration of Helsinki: ethical principles for medical research involving human subjects. JAMA. (2013) 310:2191–4. 10.1001/jama.2013.281053 24141714

[21] Rivera-Rivera P Rios-lago M Sanchez-Casarrubios S Salazar O Yus M Sanz A. Cortical plasticity catalyzed by prehabilitation enables extensive resection of brain tumors in eloquent areas. J Neurosurg. (2017) 126:1323–33. 10.3171/2016.2.JNS152485 27203145

[22] Bonnetblanc F Desmurget M Duffau H. Low grade gliomas and cerebral plasticity: fundamental and clinical implications. Med Sci. (2006) 22:389–94. 10.1051/medsci/2006224389 16597408

[23] Kornfeld S Delgado Rodríguez JA Everts R Kaelin-Lang A Wiest R Weisstanner C. Cortical reorganisation of cerebral networks after childhood stroke: impact on outcome. BMC Neurol. (2015) 15:90. 10.1186/s12883-015-0309-1 26058895 PMC4466862

[24] Mikellidou K Arrighi R Aghakhanyan G Tinelli F Frijia F Crespi S. Plasticity of the human visual brain after an early cortical lesion. Neuropsychologia. (2019) 128:166–77. 10.1016/j.neuropsychologia.2017.10.033 29100949

[25] Aşkin A Tosun A Demirdal ÜS. Effects of low-frequency repetitive transcranial magnetic stimulation on upper extremity motor recovery and functional outcomes in chronic stroke patients: a randomized controlled trial. Somatosens Mot Res. (2017) 34:102–7. 10.1080/08990220.2017.1316254 28427299

[26] Paquette C Thiel A. Rehabilitation interventions for chronic motor deficits with repetitive transcranial magnetic stimulation. J Neurosurg Sci. (2012) 56:299–306. 23111290

[27] Ribeiro H Sesterhenn RB Souza A Souza AC Alves M Machado JC. Preoperative transcranial direct current stimulation: exploration of a novel strategy to enhance neuroplasticity before surgery to control postoperative pain. A randomized sham-controlled study. PLoS ONE. (2017) 12:e0187013. 10.1371/journal.pone.0187013.eCollection.2017 29190741 PMC5708693

[28] Hadoush H Banihani SA Khalil H Al-Qaisi Y Al-Sharman A Al-Jarrah M. Dopamine, BDNF and motor function postbilateral anodal transcranial direct current stimulation in Parkinson's disease. Neurodegener Dis Manag. (2018) 8:171–9. 10.2217/nmt-2017-0048 29888648

[29] Brunoni AR Padberg F Vieira ELM Teixeira AL Carvalho AF Lotufo PA. Plasma biomarkers in a placebo-controlled trial comparing tDCS and escitalopram efficacy in major depression. Prog Neuropsychopharmacol Biol Psychiatry. (2018) 86:211–7. 10.1016/j.pnpbp.2018.06.003 29894705

[30] Yu TH Wu YJ Chien ME Hsu KS. Transcranial direct current stimulation induces hippocampal metaplasticity mediated by brain-derived neurotrophic factor. Neuropharmacology. (2019) 144:358–67. 10.1016/j.neuropharm.2018.11.012 30439417

[31] Cava C Manna I Gambardella A Bertoli G Castiglioni I. Potential role of miRNAs as theranostic biomarkers of epilepsy. Mol Ther Nucleic Acids. (2018) 13:275–90. 10.1016/j.omtn.2018.09.008 30321815 PMC6197620

[32] Costard LS Neubert V Venø MT Su J Kjems J Connolly NMC. Electrical stimulation of the ventral hippocampal commissure delays experimental epilepsy and is associated with altered microRNA expression. Brain Stimul. (2019) 12:1390–401. 10.1016/j.brs.2019.06.009 31208877

